# Evaluation of Commercial Camera-Based Solutions for Tracking Hand Kinematics

**DOI:** 10.3390/s25185716

**Published:** 2025-09-13

**Authors:** Alexander H. Sprague, Christopher Vogel, Mylah Williams, Evelynne Wolf, Derek Kamper

**Affiliations:** 1 Lampe Joint Department of Biomedical Engineering at the University of North Carolina at Chapel Hill (Chapel Hill, NC) and North Carolina State University (Raleigh, NC), Raleigh, NC 27695, USA; cmvogel@ncsu.edu (C.V.); mlwill28@ncsu.edu (M.W.); efwolf2@ncsu.edu (E.W.); dgkamper@ncsu.edu (D.K.); 2 Closed-Loop Engineering for Advanced Rehabilitation Research Core, University of North Carolina at Chapel Hill and North Carolina State University, Raleigh, NC 27695, USA

**Keywords:** hand, kinematics, motion capture, Leap Motion Controller 2, MediaPipe

## Abstract

Tracking hand kinematics is essential for numerous clinical and scientific applications. Markerless motion capture devices have advantages over other modalities in terms of calibration, set up, and overall ease of use; however, their accuracy during dynamic tasks has not been fully explored. This study examined the performance of two popular markerless systems, the Leap Motion Controller 2 (LM2) and MediaPipe (MP), in capturing joint motion of the digits. Data were compared to joint motion collected from a marker-based multi-camera system (Vicon). Eleven participants performed six tasks with their dominant right hand at three movement speeds while all three devices simultaneously captured the position of hand landmarks. Using these data, digit joint angles were calculated. The root mean squared error (RMSE) and correlation coefficient (*r*) relative to the Vicon angles were computed for LM2 and MP. LM2 achieved a lower error (*p* < 0.001, mean RMSE = 14.8°) and a higher correlation (*p* = 0.007, mean *r* = 0.58) than the MP system (mean RMSE = 22.5°, mean *r* = 0.45). Greater movement speed led to significantly higher RMSE (*p* < 0.001) and lower *r* (*p* < 0.001) for MP but not for LM2. Error was substantially greater for the proximal interphalangeal joint than for other finger joints, although *r* values were higher for this joint. Overall, the LM2 and MP systems were able to capture motion at the joint level across digits for a variety of tasks in real time, although the level of error may not be acceptable for certain applications.

## 1. Introduction

Due to the functional importance of the human hand, kinematic tracking of the digits is important for a variety of applications, including assessment and rehabilitation. Measuring joint rotation in the hand, however, is challenging because of the many degrees of freedom in the hand and the close proximity of the joints.

A number of devices have been developed for capturing hand kinematics (see [1] for a review) such as the CyberGlove [2,3], flexible bend sensors [4,5], electrogoniometers [6], and inertial measurement units [7,8]. These instruments have demonstrated high accuracy and repeatability but require placing sensors accurately and consistently on the hand, or donning a glove, which can prove difficult for clinical populations. Furthermore, these devices may be expensive, require extensive calibration, or prove challenging to use, thereby limiting home or clinical use.

Motion capture systems offer an alternative means of tracking joint angles; however, systems involving markers (whether passive or active) typically involve substantial setups and calibration. Recent advances in markerless optical tracking offer a more feasible solution [9]. Rather than employing pose estimation, as has often been performed with computer-vision techniques [1], these devices track anatomical features, or landmarks, in real time. The capabilities of these systems, however, have not been fully explored. While systems, such as the Microsoft Kinect Azure [10] and OpenPose [11], have successfully captured the movement of proximal joints, tracking more distal joints has proven difficult [9].

Thus, the goal of this study was to characterize the performance of markerless tracking for the hand. We examined two popular systems: (1) the Leap Motion Controller 2 (Ultraleap Limited, Bristol, UK) [12]; and (2) MediaPipe (Google, Inc., Mountain View, CA, USA) [13]. The Leap Motion Controller 2 (LM2) uses infrared light and cameras to track hand landmarks on the wrist and each joint of all digits. Past studies showed that the Leap Motion Controller, the predecessor to the LM2, could accurately track wrist movements [14], or finger movements if multiple units were used [15]; however, evaluations of the LM2 have been limited. MediaPipe (MP) similarly tracks hand landmarks but utilizes an RGB camera with two-dimensional data; no depth trackers are incorporated. Past assessments of MP performance have been limited in terms of static postures vs. dynamic movement [16], the landmarks evaluated [17], or the number and type of hand motions [18].

The two systems were compared with a marker-based system (Vicon, Vicon Motion Systems Ltd., Oxford, UK), integrating data from 11 cameras. The Vicon data were considered as the ground truth. Participants performed various movements of the fingers and thumb at different speeds. We hypothesized that LM2, with its depth tracking, would better capture joint angles than MP, although degradation of performance with increasing speed was expected for both systems.

## 2. Materials and Methods

### 2.1. Participants

Eleven adults (6 female/5 male; ages 24.5 ± 5.7 years—mean ± standard deviation) with no known hand or arm impairments participated in this study. Hand size, measured from the distal edge of the wrist to the tip of the middle finger, ranged from 154 to 184 mm. All subjects provided informed consent in accordance with study protocols approved by the Institutional Review Board of North Carolina State University.

### 2.2. Protocol

**Figure 1:**
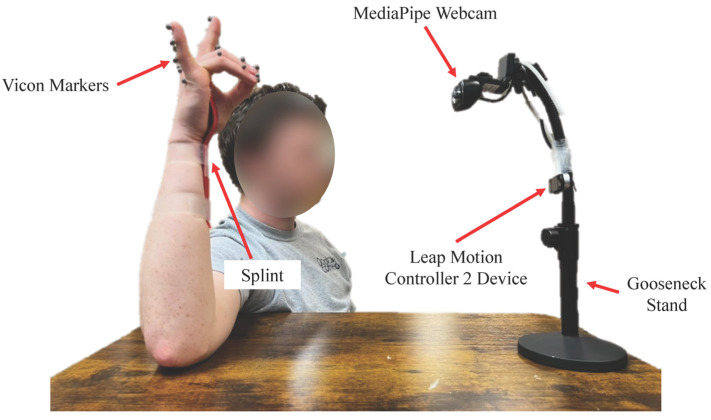
Experimental paradigm. A total of 21 6.3 mm passive markers were placed over the digit joints for tracking by the Vicon system. Cameras for LM2 and MP were positioned with the gooseneck stand to align well with the digits.

To assess the performance of the LM2 and MP systems in capturing digit kinematics, participants performed various hand motions while their hand kinematics were recorded simultaneously by three different devices: (1) LM2; (2) MP; and (3) Vicon. Participants sat in a chair with their right arm abducted and externally rotated, such that the forearm pointed upwards with the elbow flexed roughly 90°, and the palm faced forward. The upper arm was supported on a table that was adjustable in height; the wrist was splinted on the ventral side to reduce required exertion to maintain hand posture (Figure 1). Participants were instructed to minimize displacement or rotation of the hand in order to keep the hand within the field of view of all the optical systems.

For motion capture with the Vicon system, 21 passive reflective markers (6.3 mm) were taped to the wrist, finger joints, and fingertips of the right hand. For the index, middle, ring, and pinky fingers, markers were placed on the metacarpophalangeal (MCP), proximal interphalangeal (PIP), and distal interphalangeal (DIP) joints. For the thumb, markers were placed on the carpometacarpal (CMC), metacarpophalangeal (MCP), and interphalangeal (IP) joints. Before the experiment, we confirmed, through visual inspection, that this size of the Vicon marker did not interfere with LM2 or MP data capture; larger markers did seem to affect LM2 and MP data. LM2 employed a commercial dual-camera device to track the position of 22 anatomical hand landmarks without markers (the two points for the wrist were averaged). The MP system used video feed from a Logitech webcam to track the position of 21 anatomical hand landmarks. The LM2 unit and the webcam were positioned on a gooseneck support in front of the participant to optimally orient the cameras with respect to the hand.

Custom software was created using the frameworks provided for LM2 [19] (tracking service version: 6.0.0) and MP [20] (commit: e2502fd). LM2, MP, and Vicon data were recorded in parallel on each respective system. To enable data alignment, digital triggers marking the beginning of data collection were sent from the LM2 and the MP software via a serial port to an Arduino Due, which subsequently wrote the trigger signal to a digital I/O pin wired to an analog input of the Vicon system. As all components are hardwired, the Arduino operates solely to read and send the trigger signals. The Vicon records analog signals at 1 kHz; any delay introduced by triggering was assumed to be shorter than the sampling interval for each motion-tracking system. Vicon recordings were started before, and ended after, all LM2 and MP recordings to ensure the detection of trigger signals and allow for alignment of the different recordings. The motion capture sampling rate was fixed at 100 frames per second (FPS) for the Vicon and was variable for the other two systems, with LM2 and MP averaging approximately 90 and 30 FPS, respectively.

**Figure 2:**
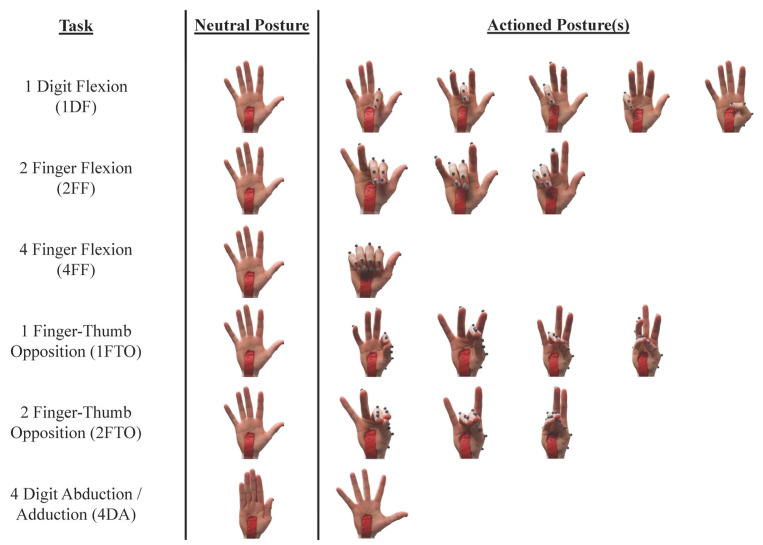
Summary of the finger and thumb movements performed by each participant: 2FF and 4FF did not include any thumb movement. For 4DA, the middle finger was intended to remain motionless.

Participants performed six hand tasks with the right hand (Figure 2): (1) single-digit flexion/extension (1DF); (2) two-finger flexion/extension (2FF); (3) four-finger flexion/extension (4FF); (4) one-finger–thumb opposition (1FTO); (5) two-finger–thumb opposition (2FTO); and (6) abduction (4DA). For each task, subjects moved between a neutral posture, with the digits extended and residing in the plane of the palm, and the final actioned posture, as dictated by the task. For the 1DF task, the participant independently flexed and extended each digit sequentially from the neutral posture to full achievable flexion while trying to minimize movement of the other digits. Participants flexed and extended pairs of adjacent fingers for 2FF while minimizing the movement of other digits. For 4FF, all four fingers were flexed and extended simultaneously. The 1FTO task involved thumb–finger opposition performed sequentially for each finger, while participants used two adjacent fingers in opposition with the thumb for 2FTO. The 4DA required maximal abduction of 4 digits with respect to the middle finger and return to adduction. These motions were selected to encompass movements of various numbers of digits (ranging from one to four) in multiple planes. Furthermore, past studies have emphasized that finger individuation [21], pinching [22], and opening/closing of the hand [22] are functionally important for daily life and valuable targets for the rehabilitation of finger movements.

Participants were instructed to complete each motion to the maximum of their comfortable range of motion while minimizing uninstructed movements. Each participant performed 5 cycles of each movement for each task (e.g., the index finger was moved from the neutral posture to maximum flexion and back 5 times in succession for the 1DF task). Participants were instructed to repeat each task at three target speeds controlled by movement frequencies of 0.5, 1.0, and 1.5 Hz, as cued with a digital metronome.

### 2.3. Analysis

LM2, MP, and Vicon data were all resampled at 30 Hz to create a fixed sampling rate for data comparison. As LM2 and MP had variable sampling rates, the timestamp of each sample was recorded and used to perform the spine interpolation involved in the resampling. All signals were then low-pass filtered forwards and backwards using a 4th-order, digital Butterworth filter with a 10 Hz cutoff frequency, considered an upper bandwidth for finger motion [23,24]. To facilitate analysis across subjects, joint angles were computed from the joint locations measured by each device using the vectors describing joint locations. Using the same joint angle calculations for each dataset, we computed angles for MCP, PIP, and DIP for each finger and MCP and IP for the thumb.

**Figure 3:**
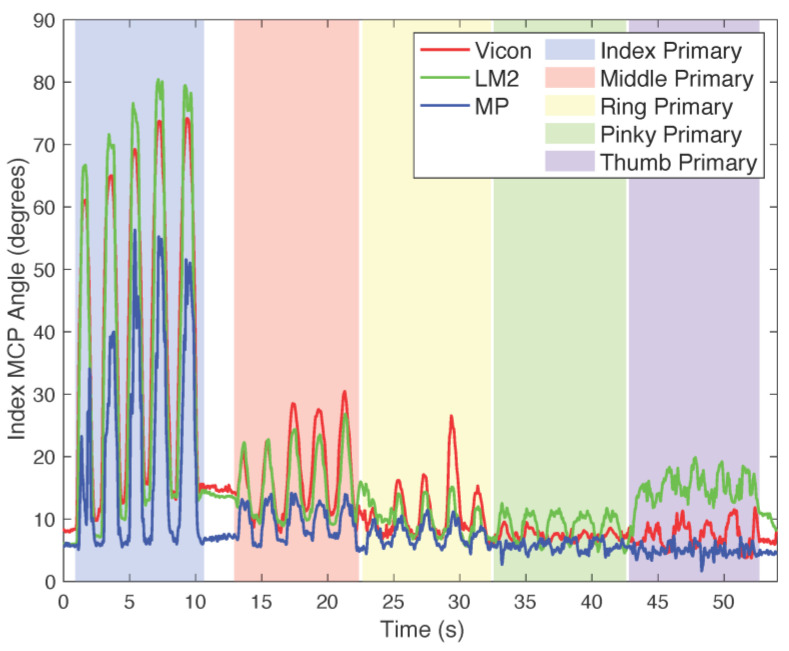
Example of data analysis for 1DF task following index MCP motion during the 0.5 Hz movement speed. Data recorded were manually divided into different, isolated time periods of instructed movement (e.g., column shaded blue) and uninstructed movement (other shaded columns). Separate analyses were performed for instructed and uninstructed movements.

Each data record for each device captured all the repetitions of a given task performed over all speeds. Thus, there were 18 data records for each subject (3 devices for 6 tasks). As we wished to distinguish tracking performance during periods when a given digit was required to move for the task from periods when it was not (and thus should have less movement), we manually separated each record into these periods (Figure 3). When the digit was instructed to move, we referred to the digit as the “primary” mover. During the instructed movement of other digits, this digit was termed “non-primary.” Primary and non-primary movements were analyzed separately.

LM2 and MP joint angles were then compared to the Vicon joint angles. The root mean squared error (RMSE) and the Pearson correlation coefficient (r) were computed over the entirety of each of the isolated periods (e.g., see shaded columns of Figure 3) for each task and speed for both the primary and non-primary conditions. As a principal analysis, we first performed repeated measures analysis of variance (rmANOVA) in SPSS (version 29.0.1.0 (171), IBM, Armonk, NY, USA) to compare the effects of device (LM2/MP) and speed, or frequency of movement (0.5/1.0/1.5 Hz), on RMSE and *r* for the primary condition across 5 tasks: 1DF, 2FF, 4FF, 1FTO, and 2FTO. We ran a separate device-speed rmANOVA for 4DA, as this task uniquely involved only MCP abduction/adduction.

For secondary analyses, we examined the effects of finger (index/middle/ring/pinky) and joint (MCP/PIP/DIP) on RMSE and *r* for both LM2 and MP for each task individually, again using only values when joints were primary. If speed were significant for LM2 or MP in the principal analysis, we analyzed speeds separately in this secondary analysis for that device. For all rmANOVAs, Mauchly’s test of sphericity was performed. In cases where the null hypothesis of sphericity was rejected, the Greenhouse–Geisser correction was used. Pairwise comparisons of estimated marginal means using a Bonferroni correction were conducted for within-subject factors that were significant.

## 3. Results

### 3.1. Data Collection

Eleven subjects successfully completed the experimental protocol. Specific tasks for some subjects were omitted due to various issues, such as synchronization failures or the hand shifting out of the field of view for one or more camera systems. Data from one participant were omitted entirely from the device-speed analysis due to missing data from four of the five tasks. Two other participants were missing data from one or two tasks but were still included in the device-speed analysis.

### 3.2. Device-Speed Analysis

**Figure 4:**
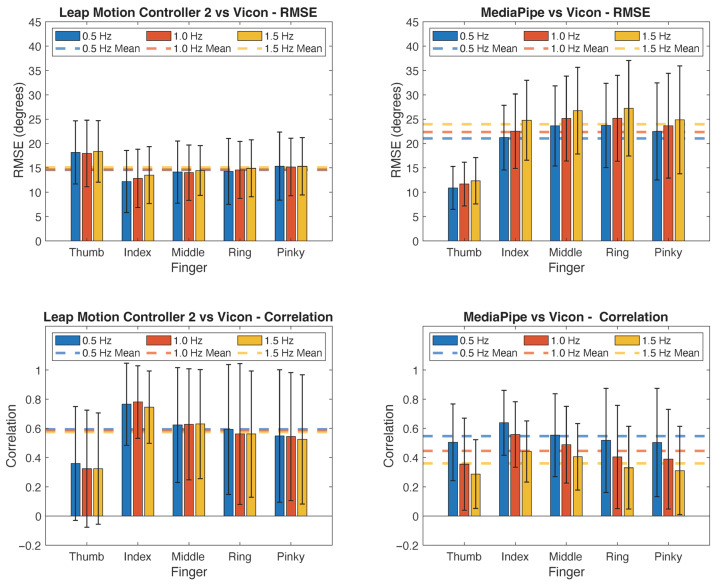
Performance of LM2 and MP across movement frequencies. LM2 (**left column**) and MP (**right column**). RMSE values (**top row**) and Pearson correlation coefficient (**bottom row**). Values averaged across all joints for a given digit and across 5 tasks (excluding 4DA). Error bars indicate standard deviation.

The principal analysis revealed that the device, speed, and the device×speed interaction all significantly impacted RMSE in the rmANOVA including 5 of the tasks (*p* < 0.001, *p* = 0.007, and *p* = 0.002, respectively). RMSE was significantly lower for LM2 (14.8° across speeds) than for MP (22.5° across speeds). The two systems also differed in terms of the impact of movement speed. While LM2 showed little impact of speed, RMSE increased with speed (movement frequency) for MP (Figure 4).

The device, speed, and the device×speed interaction significantly impacted correlation as well (*p* < 0.001, *p* < 0.001, and *p* < 0.001, respectively). Mean *r* values were significantly higher for LM2 (0.58 across all speeds) than for MP (0.45 across all speeds). The correlation generally decreased with speed, particularly for MP (ranging from 0.55 at 0.5 Hz to 0.36 at 1.5 Hz).

For the 4DA task, neither the device, nor the speed, nor the device×speed interaction significantly affected RMSE (*p* = 0.088, *p* = 0.851, *p* = 0.152, respectively). The mean RMSE was 9.5° for LM2 and 8.2° for MP. The speed and device×speed interaction did significantly affect *r* (*p* < 0.001, *p* = 0.002, respectively), but device did not (*p* = 0.63). The mean correlation was 0.52 for LM2 and 0.49 for MP. The correlation was lower at higher speeds, particularly for MP, with *r* = 0.56, *r* = 0.51, and *r* = 0.48 for LM2 and *r* = 0.61, *r* = 0.51, and *r* = 0.36 for MP at 0.5, 1.0, and 1.5 Hz, respectively.

**Figure 5:**
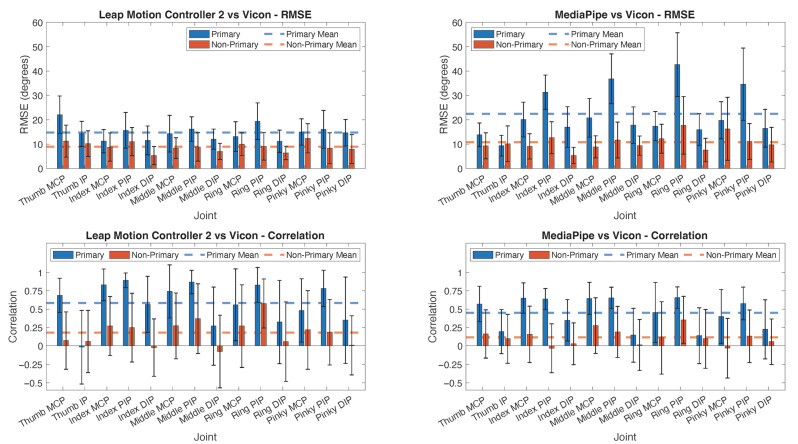
Performance of Leap Motion Controller 2 (LM2) and MediaPipe (MP) when involved in an intended motion (primary, blue bar) and when not involved in an intended motion (non-primary, red bar) for different joints. LM2 (**left column**) and MP (**right column**). RMSE values (**top row**) and Pearson correlation coefficient (**bottom row**). Values averaged across all speeds and across five tasks (excluding 4DA). Error bars indicate standard deviation.

For non-primary movers, RMSE values for both LM2 and MP were substantially smaller than for primary movers: RMSE =9.0 ± 1.9° for LM2 and RMSE = 10.8° ± 3.2° for MP for non-primary movers (Figure 5). The correlation values, however, decreased substantially for non-primary users: *r* = 0.18 ± 0.18 for LM2 and *r* = 0.12 ± 0.11 for MP.

### 3.3. Finger-Joint Analysis

Secondary analyses were conducted to determine the impact of the joint and finger on RMSE and correlation. As speed impacted values for MP, statistical tests were run separately for each speed. The rmAMOVA results revealed that the joint was significant for all tasks and speeds for the RSME for both LM2 and MP, except 2FTO for the LM2. The finger×joint interaction was significant for the RMSE for all tasks, aside from 2FF at 1.0 Hz and 4FF for all speeds for MP. The finger generally did not have a statistically significant effect on RMSE.

Errors tended to be greatest for the PIP joints (Figure 5). Pairwise comparisons of estimated marginal mean RMSE values for joints showed that MCP was significantly different than PIP for all 1DF, 2FF, and 4FF tasks for both LM2 and MP at all speeds, except for 2FF for LM2. Additionally, the RMSE for PIP was significantly different than DIP for all tasks and speeds, except for 2FF and 2FTO for LM2. For the MP system, the mean RMSE exceeded 40° for the ring PIP. In contrast, the MCP and DIP mean RMSE values were generally below 20°.

In terms of correlation, the joint significantly impacted *r* for LM2 for the 4FF, 1FTO, and 2FTO tasks and for MP across all tasks and all speeds, except 4FF at 0.5 Hz and 1.0 Hz. The finger×joint interaction generally did not have a significant impact on r. Pairwise comparisons revealed that PIP correlation values were significantly greater than DIP values for all tasks at all speeds, except 1FTO at 1.5 Hz for MP. Thumb IP correlation values were especially low for both systems (Figure 5).

### 3.4. Task Analysis

**Figure 6:**
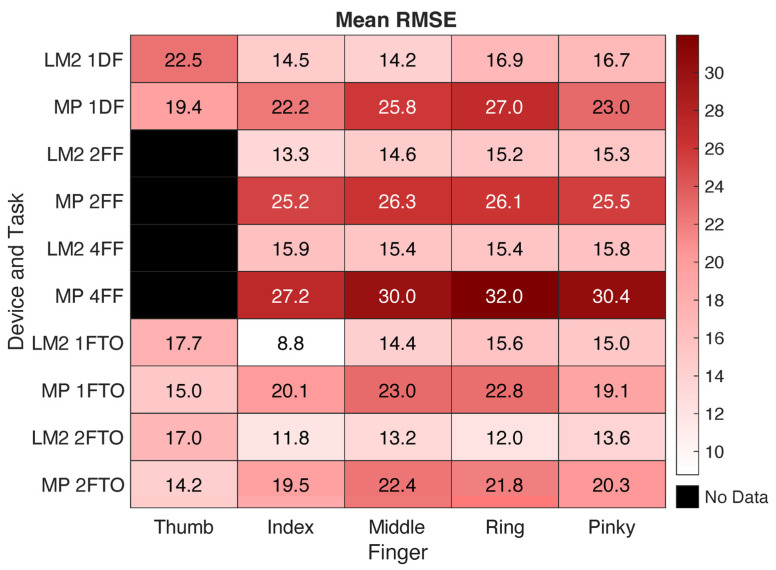
Matrix showing average RMSE values across five selected tasks for each finger and each device. Values averaged across speeds. Darker red indicates greater RMSE.

**Figure 7:**
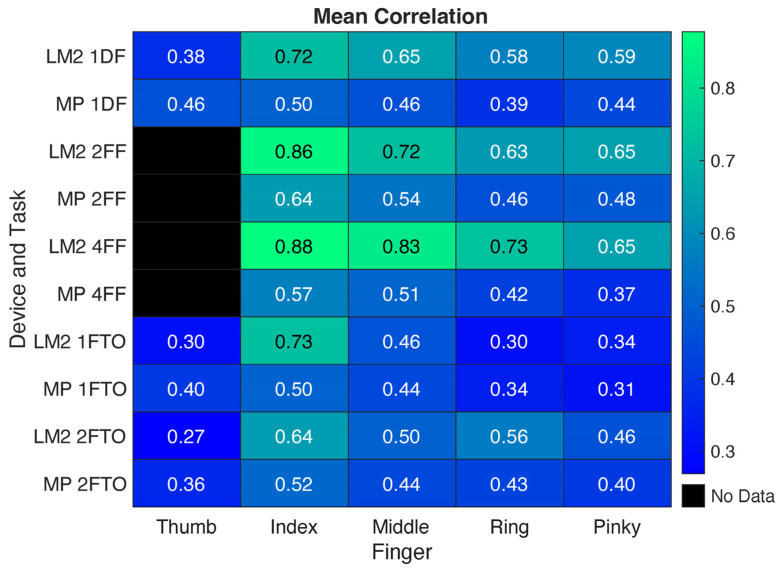
Matrix showing mean correlation coefficients across tasks and devices for each digit. Green coloration indicates higher correlation.

In terms of RMSE, LM2 tended to produce relatively consistent results across tasks. The mean RMSE was slightly higher for 1DF, 2FF, and 4FF than for 1FTO and 2FTO, especially for the index finger and thumb; however, these differences were within 6° (Figure 6). MP similarly produced smaller errors during the 1FTO and 2FTO movements, with even greater differences with the 1DF, 2FF, and 4FF tasks (more than 10° in some cases). The correlations, however, tended to be greatest for the 2FF and 4FF tasks. As shown in Figure 7, LM2 had the highest mean correlation across fingers for 4FF and the lowest for 1FTO. MP had the highest mean correlation for 2FF and the lowest also for 1FTO.

## 4. Discussion

This study evaluated the performance of two prominent commercial devices for markerless hand tracking (Leap Motion Controller 2 and MediaPipe). In general, the markerless systems were able to track the movement of individual joints, albeit with greater error than reported for other sensors placed on the hand.

The mean RMSE across all joints for the five tasks involving primarily joint flexion and extension were 14.8° for LM2 and 22.5° for MP. In comparison, RMSE values typically under 10° were reported with the CyberGlove III over both static and dynamic tasks with subject-specific calibration methods [25], although RMSE increased by approximately 5–10° when calibrations were not specific to each subject. Errors of no more than 7.5° were reported for dynamic movements with an IMU-based sensor [8]. Both LM2 and MP more accurately captured pure MCP abduction/adduction, with lower mean RMSE values (below 10°) for the 4DA task, although mean *r* values (around 0.50) were similar to correlation values for other tasks. These RMSE values are within a few degrees of the errors reported for abduction/adduction when using IMU-based sensors [7].

Overall, the LM2 output better matched values from the multi-camera Vicon system than MP. RMSE was significantly lower and the correlation with the Vicon data was significantly higher for LM2. The depth tracking provided by the two infrared cameras may have led to better results [18]. The RMSE was lower across digits and tasks for LM2, except for the thumb. As the thumb was largely moving in a plane oblique to the planes of movement for the digits, this motion may have been somewhat more difficult to detect. The LM2 had especially strong correlations with Vicon data for the index finger (r = 0.88) for the four-finger flexion task, suggesting that it detected the motion of this finger very well. The location of the index finger, on the outside of the four fingers and adjacent to the distinctive thumb, may have aided tracking. The inclusion of more cameras may have improved performance of the MP system [26,27]; however, this would require a more involved set-up that reduces feasibility for home use.

When a digit was instructed to move, its joints tended to have higher RMSE but also higher correlation than when it was not instructed to move. This may be partly explained by a relationship between RMSE and total displacement. Indeed, one group introduced a measure termed percent root mean square error (PRMSE) that sought to normalize RMSE in terms of the amplitude of joint rotation [18]. As the amount of rotation of instructed joints (primary) in our study was higher than that of non-instructed joints (non-primary), PRMSE may be a useful metric for future investigations directly comparing these conditions. Conversely, the lack of range seemed to lower correlation values, which averaged less than 0.20 for both LM2 and MP for non-primary movers. This suggests that neither device tracked the subtle unintentional movements well, which may be an issue when precisely quantifying individuation. Seemingly, below a certain threshold of joint displacement, neither device captured movement well, although the absolute error was low.

In agreement with past studies of physical sensors, such as IMUs [8], speed significantly degraded the performance of the MP system, both in terms of increasing error and decreasing correlation. This contrasts with a previous study of MP that found that RMSE decreased with increased speed [18]. As that study employed faster movement frequencies (approximately 1.2, 1.9, and 2.3 Hz) and some different hand tasks, direct comparison with our results is difficult. In contrast to MP, the LM2 data showed little effect of speed on either RMSE or correlation.

Secondary analyses suggested that tracking performance varied across joints. RMSE was higher for the PIP joints than for the MCP and DIP joints across tasks, especially for the tasks involving pure flexion and extension (1DF, 2FF, and 4FF). This occurred despite the fact that the magnitude of MCP flexion was similar to that of PIP flexion across tasks. The greater RMSE associated with tracking the PIP joint may have arisen from greater difficulty in locating the proximal and middle segments involved in PIP angle calculation as compared to the hand and distal segment used as part of the MCP and DIP angle calculations, respectively. The finger×joint interaction was often significant; the error for the PIP joint of the ring finger was especially high for both systems and is worthy of attention. These tracking errors may have arisen from the ring finger being surrounded by other fingers, the proximity of the pinky DIP joint to the ring PIP joint, or the tendency for the ring finger to move in conjunction with other digits, which could bias the motion-training sets used by the systems. PIP correlation values, however, were quite strong across tasks and tended to be at least as large as MCP correlation values. Correlation coefficients for PIP and DIP were significantly different for almost all tasks, with DIP correlations being smaller. Thus, PIP motion was often properly detected, but not quantified, while the smaller DIP motions were more difficult for the systems to detect.

In terms of task-specific performance, LM2 had higher correlations for the digit flexion/extension tasks than for the finger–thumb opposition tasks, although RMSE was slightly higher (2–3°) for the former. MP had substantially higher errors for the flexion/extension tasks than for the thumb–finger opposition tasks. The errors exceeded 30° for some digits.

Generalization of these results is limited to the specific digit motions and relative viewing angles of each device used here. We attempted to optimize the location and orientation of each of the camera systems with respect to the hand. Other camera postures, relative to the hand, may lead to degradation in overall tracking performance but may be better suited for tracking movements in specific planes. Furthermore, while this study evaluated the use of a single unit or camera, the integration of multiple devices, as demonstrated in [15], may improve performance by providing more viewing angles and potentially leading to better tracking in various planes of movement. This could result in the improved capturing of fine movements of both intended and unintended movers, which may be particularly useful in clinical assessments. However, the multiple-camera arrangements could also significantly increase the effort required to set up and calibrate the system for each use and increase the complexity of data analysis.

The sample size included in this study (*n* = 11) was relatively small, although hand size was varied. In future studies, building upon this preliminary assessment, it would be valuable to increase sample size and focus on older adults and members of clinical populations, who are likely to be the targeted participants for clinical assessments and rehabilitation. While participants had a variety of skin tones, we did not control for this and so did not evaluate the impact of skin tone on motion capture by these systems. Lighting conditions were consistent for all subjects but could be an area for future investigation. This is especially relevant for home environments with variable lighting.

We evaluated temporal accuracy of LM2 and MP by calculating RMSE and correlation values, in comparison to Vicon, over the entirety of selected tasks. Temporal shifts between the Vicon and the LM2 and MP data recordings may lead to higher RMSE and lower correlation values. The aforementioned triggering system was implemented to enable data alignment of the recorded position data of key landmarks. Joint angles were computed post hoc for all three systems. Still, temporal shifts due to misalignment or data processing performed by each system may have affected absolute results. We would not anticipate, however, a significant impact on relative results across digits and joints.

It is also possible that the splint may have hindered natural movement of the thumb and pinky finger, especially during opposition, although no participants reported any discomfort or inability to fully perform any task motions. As all devices simultaneously captured the same motion, the presence of the splint should not have affected the device comparison, although it may affect the generalization of results to specific tasks. Lastly, we are assuming that the Vicon data represented ground truth. Further investigation is warranted to examine each of these potential limitations.

## 5. Conclusions

In conclusion, the Leap Motion Controller 2 and MediaPipe were generally able to track digit joint movement, albeit with the noted issues related to accuracy, particularly for the PIP joint. LM2 performed better than MP, especially with respect to speed. MP exhibited degradation in quantifying motion at higher speeds, while LM2 did not. To address these limitations, future work could include testing LM2 and MP with more tasks and conditions, hand sizes and shapes, and viewing angles. Furthermore, a test of practical implementation could be conducted by implementing one or both of these devices in a clinical or home setting and garnering user feedback.

## Data Availability

The de-identified data presented in this study can be made available upon reasonable request to the corresponding author.

## References

[1] Chen W., Yu C., Tu C., Lyu Z., Tang J., Ou S., Fu Y., Xue Z. (2020). A Survey on Hand Pose Estimation with Wearable Sensors and Computer-Vision-Based Methods. Sensors.

[2] Jack D., Boian R., Merians A.S., Tremaine M., Burdea G.C., Adamovich S.V., Recce M., Poizner H. (2001). Virtual Reality-Enhanced Stroke Rehabilitation. IEEE Trans. Neural Syst. Rehabil. Eng..

[3] Conway B.J., Taquet L., Boerger T.F., Young S.C., Krucoff K.B., Schmit B.D., Krucoff M.O. (2023). Quantitative Assessments of Finger Individuation with an Instrumented Glove. J. Neuroeng. Rehabil..

[4] Saggio G., Pallotti A., Sbernini L., Errico V., Paolo F. (2016). DI Sensors & Transducers Feasibility of Commercial Resistive Flex Sensors for Hand Tracking Applications. Sens. Transducers.

[5] Simone L.K., Kamper D.G. (2005). Design Considerations for a Wearable Monitor to Measure Finger Posture. J. Neuroeng. Rehabil..

[6] McHugh B.P., Morton A.M., Akhbari B., Molino J., Crisco J.J. (2020). Accuracy of an Electrogoniometer Relative to Optical Motion Tracking for Quantifying Wrist Range of Motion. J. Med. Eng. Technol..

[7] Salchow-Hömmen C., Callies L., Laidig D., Valtin M., Schauer T., Seel T. (2019). A Tangible Solution for Hand Motion Tracking in Clinical Applications. Sensors.

[8] Shenoy P., Gupta A., Varadhan S.K.M. (2022). Design and Validation of an IMU Based Full Hand Kinematic Measurement System. IEEE Access.

[9] Lam W.W.T., Tang Y.M., Fong K.N.K. (2023). A Systematic Review of the Applications of Markerless Motion Capture (MMC) Technology for Clinical Measurement in Rehabilitation. J. Neuroeng. Rehabil..

[10] Brambilla C., Marani R., Romeo L., Nicora M.L., Storm F.A., Reni G., Malosio M., D’Orazio T., Scano A. (2023). Azure Kinect Performance Evaluation for Human Motion and Upper Limb Biomechanical Analysis. Heliyon.

[11] Stenum J., Rossi C., Roemmich R.T. (2021). Two-Dimensional Video-Based Analysis of Human Gait Using Pose Estimation. PLoS Comput. Biol..

[12] El Chemaly T., Neves C.A., Fu F., Hargreaves B., Blevins N.H. (2024). From Microscope to Head-Mounted Display: Integrating Hand Tracking into Microsurgical Augmented Reality. Int. J. Comput. Assist. Radiol. Surg..

[13] Güney G., Jansen T.S., Dill S., Schulz J.B., Dafotakis M., Antink C.H., Braczynski A.K. (2022). Video-Based Hand Movement Analysis of Parkinson Patients before and after Medication Using High-Frame-Rate Videos and MediaPipe. Sensors.

[14] Smeragliuolo A.H., Hill N.J., Disla L., Putrino D. (2016). Validation of the Leap Motion Controller Using Markered Motion Capture Technology. J. Biomech..

[15] Houston A., Walters V., Corbett T., Coppack R. (2021). Evaluation of a Multi-Sensor Leap Motion Setup for Biomechanical Motion Capture of the Hand. J. Biomech..

[16] Budzinski C., Wu H.-L., Sarraf E., Miller S., Moore J. Accuracy of mediapipe visual hand tracking for use in medical training procedures. Proceedings of the 2024 Design of Medical Devices Conference.

[17] Chunduru V., Roy M., Romit N.S.D., Chittawadigi R.G. (2021). Hand Tracking in 3D Space Using MediaPipe and PnP Method for Intuitive Control of Virtual Globe. Proceedings of the IEEE Region 10 Humanitarian Technology Conference (R10-HTC).

[18] Amprimo G., Masi G., Pettiti G., Olmo G., Priano L., Ferraris C. (2024). Hand Tracking for Clinical Applications: Validation of the Google MediaPipe Hand (GMH) and the Depth-Enhanced GMH-D Frameworks. Biomed. Signal Process. Control.

[19] Ultraleap Leap Motion Controller 2 Downloads. https://www.ultraleap.com/downloads/leap-motion-controller-2/.

[20] Nicholas Renotte (GitHub Username: Nicknochnack) MediaPipeHandPose. https://www.github.com/nicknochnack/MediaPipeHandPose.

[21] McCall J.V., Hu X., Kamper D.G. (2023). Exploring Kinetic and Kinematic Finger Individuation Capability in Children with Hemiplegic Cerebral Palsy. Percept. Mot. Ski..

[22] McConnell A.C., Moioli R.C., Brasil F.L., Vallejo M., Corne D.W., Vargas P.A., Stokes A.A. (2017). Robotic Devices and Brain-Machine Interfaces for Hand Rehabilitation Post-Stroke. J. Rehabil. Med..

[23] Stiles R.N., Randall J.E. (1967). Mechanical Factors in Human Tremor Frequency. J. Appl. Physiol..

[24] Heimhofer C., Neumann A., Odermatt I., Bächinger M., Wenderoth N. (2024). Finger-Specific Effects of Age on Tapping Speed and Motor Fatigability. Front. Hum. Neurosci..

[25] Heinrich S., Michaelis J., Reiher I., Coppers B., Lohmayer M., Fleischmann E., Kleyer A., Schett G., De Craemer A.S., Elewaut D. (2024). Comparison and Improvement of CyberGlove III Calibration Methods. IEEE Sens. J..

[26] Li Z., Kanazuka A., Hojo A., Nomura Y., Nakaguchi T. (2025). Multi-Metric Assessment of Hand Motion with Multi-Camera for Puncture Technique Training. Measurement.

[27] Mulla D.M., Majoni N., Tilley P.M., Keir P.J. (2025). Two Cameras Can Be as Good as Four for Markerless Hand Tracking During Simple Finger Movements. J. Biomech..

